# Closure with a dual-action tissue clip for full-thickness defects following endoscopic submucosal dissection of a lesion involving a colonic diverticulum

**DOI:** 10.1055/a-2749-2818

**Published:** 2025-12-08

**Authors:** Ryosuke Kobayashi, Kingo Hirasawa, Atsushi Sawada, Masafumi Nishio, Chiko Sato, Shin Maeda

**Affiliations:** 1 26437 Division of Endoscopy, Yokohama City University Medical Center, Yokohama, Japan; 2 26438 Department of Gastroenterology, Yokohama City University School of Medicine Graduate School of Medicine, Yokohama, Japan

Although endoscopic submucosal dissection (ESD) is widely accepted for colorectal tumors, ESD of tumors involving a diverticulum remains a challenge. In particular, the absence of a muscle layer in diverticula makes intraoperative perforation almost inevitable 1 2 . Thus, reliable closure after ESD is crucial. Here, we report a case in which complete closure was achieved using a dual-action tissue (DAT) clip for a full-thickness defect after ESD of a tumor involving a colonic diverticulum. An 18 mm protruding lesion extended into a diverticulum in the ascending colon ( Fig. 1 **a** ). ESD was performed under conscious sedation and using carbon dioxide insufflation. A small caliber transparent hood (DH-29CR; Fujifilm, Tokyo, Japan) was attached to the tip of the endoscope, and a 1.5-mm ESD knife (T-type Cutting Knife; Micro-Tech, Nanjing, China) was used for ESD. The water pressure technique was used to dissect the submucosa, and a multi-loop device was used for traction. Submucosal dissection was performed after completing the circumferential incision. When encountering the lesion extending into the diverticulum, a short myotomy was performed in front of the diverticulum to create a gap to enter the space below the diverticulum 3 . The short myotomy was followed by a muscular incision around the lesion, and the tissue beneath the diverticulum was resected ( Fig. 1 **b, c** ). The lesion was resected in one piece. Following the completion of ESD, the full-thickness defect was closed using a DAT clip and ordinary endoscopic clips. The muscle layer was inverted and tightly closed using a DAT clip, resulting in complete defect closure ( Fig. 1 **d–f** ; Video 1 ). The patient was discharged on postoperative day 3. A DAT clip enabled muscle layer inversion, allowing for secure and reliable layered closure using endoscopic clips.

**Fig. 1:**
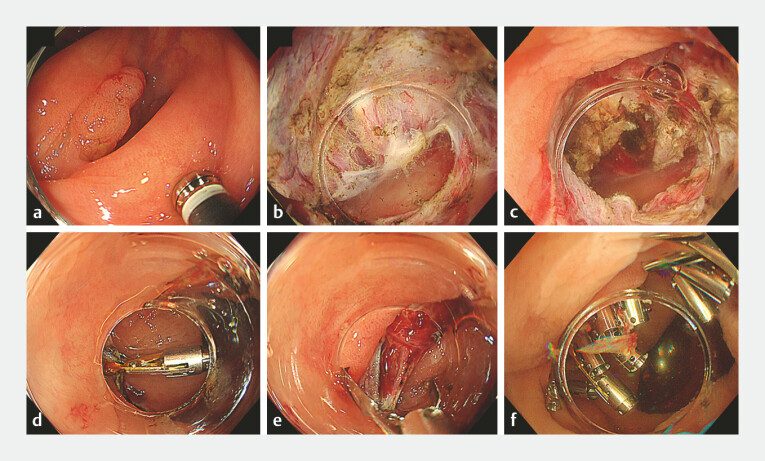
**a** The tumor involves a colonic diverticulum. **b** The space beneath the diverticulum is visualized. **c** The full-thickness defect is seen after endoscopic submucosal dissection. **d** The dual-action tissue clip is placed first. **e** The muscle layer is inverted using a dual-action tissue clip to facilitate defect closure. **f** The defect is closed completely.

Dual-action clip closure for full-thickness defects following endoscopic submucosal dissection of a lesion involving a colonic diverticulum.Video 1

Endoscopy_UCTN_Code_CPL_1AJ_2AD_3AD

## Notes

Endoscopy E-Videos https://eref.thieme.de/e-videos
*E-Videos* is an open access online section of the journal *Endoscopy* , reporting on interesting cases and new techniques in gastroenterological endoscopy. All papers include a high-quality video and are published with a Creative Commons CC-BY license. Endoscopy E-Videos qualify for HINARI discounts and waivers and eligibility is automatically checked during the submission process. We grant 100% waivers to articles whose corresponding authors are based in Group A countries and 50% waivers to those who are based in Group B countries as classified by Research4Life (see: https://www.research4life.org/access/eligibility/ ). This section has its own submission website at https://mc.manuscriptcentral.com/e-videos .

## References

[1] Ikezawa N Toyonaga T Tanaka S Feasibility and safety of endoscopic submucosal dissection for lesions in proximity to a colonic diverticulum Clin Endosc 2022 55 417 425 10.5946/ce.2021.245 35545214 PMC9178129

[2] Masunaga T Kato M Sasaki M Colorectal endoscopic submucosal dissection using the water pressure method for diverticulum-associated lesions: A case series study (with video) Endosc Int Open 2023 11 E305 E314 10.1055/a-1961-1800 37025155 PMC10072927

[3] Kobayashi R Hirasawa K Maeda S Novel technique “short myotomy” during endoscopic submucosal dissection for a diverticulum-associated colonic lesion Dig Endosc 2025 37 211 213 10.1111/den.14941 39444060

